# Multiplex one-step Real-time PCR by Taqman-MGB method for rapid detection of pan and H5 subtype avian influenza viruses

**DOI:** 10.1371/journal.pone.0178634

**Published:** 2017-06-02

**Authors:** Zhujun Zhang, Dong Liu, Wenqiang Sun, Jing Liu, Lihong He, Jiao Hu, Min Gu, Xiaoquan Wang, Xiaowen Liu, Shunlin Hu, Sujuan Chen, Daxin Peng, Xiufan Liu

**Affiliations:** 1 Animal Infectious Disease Laboratory, School of Veterinary Medicine, Yangzhou University, Yangzhou, China; 2 Jiangsu Co-innovation Center for Prevention and Control of Important Animal Infectious Diseases and Zoonosis, Yangzhou University, Yangzhou, China; 3 Key Laboratory of Prevention and Control of Biological Hazard Factors (Animal Origin) for Agrifood Safety and Quality, Ministry of Agriculture of China, Yangzhou University (26116120), Yangzhou, China; Sun Yat-Sen University, CHINA

## Abstract

Avian influenza virus (AIV) can infect a variety of avian species and mammals, leading to severe economic losses in poultry industry and posing a substantial threat to public health. Currently, traditional virus isolation and identification is inadequate for the early diagnosis because of its labor-intensive and time-consuming features. Real-time RT-PCR (RRT-PCR) is an ideal method for the detection of AIV since it is highly specific, sensitive and rapid. In addition, as the new quencher MGB is used in RRT-PCR, it only needs shorter probe and helps the binding of target gene and probe. In this study, a pan-AIV RRT-PCR for the detection of all AIVs and H5-AIV RRT-PCR for detection of H5 AIV based on NP gene of AIV and HA gene of H5 AIV were successfully established using Taqman-MGB method. We tested 14 AIV strains in total and the results showed that the pan-AIV RRT-PCR can detect AIV of various HA subtypes and the H5-AIV RRT-PCR can detect H5 AIV circulating in poultry in China in recent three years, including H5 viruses of clade 7.2, clade 2.3.4.4 and clade 2.3.2.1. Furthermore, the multiplex detection limit for pan-AIV and H5-AIV RRT-PCR was 5 copies per reaction. When this multiplex method was applied in the detection of experimental and live poultry market samples, the detection rates of pan-AIV and H5 AIV in RRT-PCR were both higher than the routine virus isolation method with embryonated chicken eggs. The multiplex RRT-PCR method established in our study showed high sensitivity, reproducibility and specificity, suggesting the promising application of our method for surveillance of both pan AIV and prevalent H5 AIV in live poultry markets and clinical samples.

## Introduction

Avian Influenza Virus (AIV) infection in poultry can lead to various symptoms, including growth retardation [1, 2], respiratory signs [1, 3] and decreased egg production[4]. Human beings and other animals also can be infected by AIV [5–8] which consists of 16 HA (Hemagglutinin, HA) subtypes and 9 NA (Neuraminidase, NA) subtypes [9]. Among these subtypes, the H5 AIV has been a substantial threat to public health as WHO reported 858 people infected by H5N1 while 453 people died by March 2017 [10]. Moreover, H5N1 and H5NX viruses of clade 7.2, clade 2.3.2.1c and 2.3.4.4 which occurred through mutation and recombination currently with other N2, N6 and N8 influenza viruses in poultry [11] have been the most prevalent H5 viruses in China [2, 12, 13], Viet Nam, Laos [14] and other countries [15].

Until now, the gold standard method of identifying AIV and its subtypes encouraged by OIE (Office International Des Epizooties, OIE) is virus isolation with embryonated chicken eggs before hemagglutination assay (HA) and hemagglutination inhibition test (HI) [16]. However, this traditional method is time-consuming and thus cannot be used as a rapid diagnostic technology. Other methods such as gold immunochromatographic assay [17], microarray [18, 19], immunosensor [20], immune-fluorescence [21] and enzyme linked immunosorbant assay [22–25] are also used in AIV detection. However, some of them fail to subtype AIV while others cannot be applied in early diagnosis owing to inadequate sensitivity [17, 26–28].

Real-time reverse transcriptase/polymerase chain reaction (RRT-PCR) [29] is a method with high specificity as well as high sensitivity. Taqman-MGB (minor groove binding, MGB) probe is a new probe firstly reported in 2000 [30]. This probe replaces traditional Tamra at 3’end with MGB as a quencher. Recently, Taqman-MGB probes are widely applied in pathogen detection and single nucleotide polymorphism (SNP) diagnosis. E.g., Taqman-MGB probes were used in the detection of equine herpes virus 5 (EHV-5) [31], infectious bursal disease virus (IBDV) [32], clostridium piliforme [33], coliforms [34], trisomy 21 [35] and differentiation of virulent and vaccine strains of avian paramyxovirus type 1 [29]. However, as far as we know, there are no reports associated with Taqman-MGB probe that targeting NP (nucleoprotein) gene of AIV despite of its high conservation. Additionally, no article about Taqman-MGB probe for the detection of multiple H5 clades was ever found although virus variation has occurred frequently.

In this study, in order to efficiently and simultaneously detect pan-AIV and prevalent H5 clades, two Taqman-MGB probes targeting AIV-NP and H5-HA fragments were designed respectively and used in reaction simultaneously. Our study suggest that the multiplex RRT-PCR method established in this study has a higher sensitivity than traditional virus isolation and can be used in live poultry markets for surveillance of pan-AIV and prevalent H5 clades.

## Materials and methods

### Ethics statement

This study was carried out in strict accordance with the recommendations in the Guide for the Care and Use of Laboratory Animals of the Ministry of Science and Technology of the People’s Republic of China. The protocols for animal experiments were approved by Jiangsu Administrative Committee for Laboratory Animals (approval number: SYXK-SU-2007-0005), and complied with the guidelines of Jiangsu laboratory animal welfare and ethics of Jiangsu Administrative Committee of Laboratory Animals. All efforts were made to minimize suffering and to reduce the number of animals used.

### Animals

Ten male 6-weeks-old SPF chickens were purchased from Weike Technology Co. (Yangzhou, China). All experiments involving live viruses and animals were performed in negative-pressure isolators with HEPA filters in a biosafety level 3 (BSL3) animal facilities in accordance with the institutional biosafety manual. Animal suffering was minimized by providing free access to food and water. Moreover, the temperature was between 20–25°C and a 12-h light/dark cycle was kept. During experiment, animals were monitored by laboratory staff twice a day and there were no animal illnesses or unexpected deaths. Animals were euthanized with sodium pentobarbital after experiments.

### Preparation of H5-HA and NP plasmids

For quantitative standard plasmids, we constructed two plasmids targeting NP gene and HA gene respectively. NP and HA genes were originated from a previously reported strain QD5 (Accession Number, KT221066-KT221067) [2], which was a H5N8 AIV of clade 2.3.4.4 isolated in 2014. They were amplified with universal primer sets of NP and HA gene [36] with BsmB I restriction sites (New England Biolabs, Beijing, China) and then cloned into PHW2000 vectors. The nucleotides of plasmids were sequenced (GenScript, Nanjing, China) and purified with a QIAprep Spin Miniprep Kit (Qiagen, Hilden, Germany).

### Viruses

The H5 subtype AIV of different clades and H1, H3, H4, H6, H7, H8, H9, H10, H11 subtype AIVs (**Table 1**) were isolated from 2013 to 2016 in our laboratory and amplified in the allantoic cavities of 10-day-old SPF chicken eggs. All gene accession numbers are available from NCBI database (KY472791~KY472797, KY486469-KY486470, EF061123, KT221082, KY437800, KY437768 and KY437776). In addition, Newcastle disease viruses (NDV), infectious bursal disease virus (IBDV), infectious bronchitis virus (IBV) and Marek’s disease virus (MDV) were also provided by our lab to serve as negative controls.

**Table 1:** AIV strains used in this study and the detection limit of RRT-PCR method.

AIV Strain	Subtype	EID_50_ ^*a*^	Detection limit ^*b*^
A/duck/Shandong/SDd11/2013	H1N1	10^−8.625^	0.042
A/duck/Jiangsu/YZD3/2013	H3N2	10^−8.0^	0.1
A/duck/Anhui/AHd38/2014	H4N6	10^−7.625^	0.42
A/goose/Yangzhou/0420/2014	H5N8, 2.3.4.4	10^−7.33^	0.021
A/duck/Beijing/BJ7/2014	H5N2, 7.2	10^−6.625^	0.42
A /chicken/Eastern China/1404/2014	H5N1, 2.3.2.1c	10^−6.625^	0.042
A/chicken/Yangzhou/YJD/2014	H5N6, 2.3.4.4	10^−8.0^	0.1
A/goose/Yangzhou/0403/2014	H5N1, 2.3.2.1c	10^−8.33^	0.021
A/duck/Jiangsu/119/2015	H6N2	10^−5.67^	0.046
A/Chicken/Zhejiang/JX164/2015	H7N9	10^−7.65^	0.044
A/duck/Yangzhou/02/2005	H8N4	10^−6.67^	0.046
A/Chicken/Eastern China/0923/2015	H9N2	10^−8.167^	0.14
A/duck/Jiangsu/XZD53/2014	H10N7	10^−6.5^	0.32
A/duck/Jiangsu/YZD1/2013	H11N9	10^−8.0^	1.0

a, EID_50_/0.1mL

b, Detection limit of RRT-PCR (EID_50_)

Reed-Muench method [16] is commonly used in the calculation of 50% lethal dose or 50% infectious dose to measure the virulence of a toxin or pathogen. To determine the EID_50_ (50% embryo infectious dose), 0.1mL of 10-fold serially diluted virus aliquots are inoculated in embryonated eggs. After 3–4 days, embryo allantoic liquid was collected for HA titration and the 50% infectious dose of the viruses were further calculated using the Reed-Muench method [16].

### Extraction of viral nucleic acids

High Pure Viral Nucleic Acid Kit (Roche Molecular Biochemicals, Indianapolis, IN, USA) was used to extract viral nucleic acids in allantoic fluids or antibiotics-containing PBS (penicillin 2000 unit/mL, streptomycin 10 mg/mL, gentamycin 250 μg/mL, kanamycin, 250 μg/mL). Briefly, 200μL samples were incubated with Proteinase K at 72°C for 10 minutes. After centrifugation, viral nucleic acids were purified with inhibitor removal buffer and then with wash buffer in filter columns with collection tubes. Finally, nucleic acids were eluted in 20–30 μL elution buffer and stored at -70°C for RRT-PCR.

### One-step Real-time PCR for detection of H5 subtype AIV and pan-AIV

#### Design of probe and primers

Sequences of H5-HA and AIV-NP gene from 2005 to 2015 were obtained from GenBank database. Clustal W alignments of H5-HA and NP multiple sequences were performed in MEGA 5.0 software before conserved domains were selected for the design of primers and probes. To detect H5 subtype AIV and pan-AIV, gene-specific probes and primers of H5-HA and AIV-NP were designed respectively using ABI Primer Express 3.0.1 and further tested by Oligo 7.0 software. Then the specificity of two sets of primers and probes were verified in Blastn search engine (http://www.ncbi.nlm.nih.gov/). The primers and probes used in this study were listed in **Table 2**. Both probes were modified with MGB at 3’-end.

**Table 2:** Primers and probes used in this study.

Name	Sequence (5’-3’)	Position	Strand
**H5-HA FP**	CTTGCGACTGGGCTCAGAAAT	985–1005	Sense
**H5-HA RP**	TTTGGGTGGATTCTTTGTCTGC	1141–1163	Antisense
**H5-HA P**	VIC-CATTCCTTGCCATCC-MGB	1071–1086	Antisense
**NP FP**	ACCAGAAGATKTGTCMTTCCAGGG	1369–1393	Sense
**NP RP**	TACTCCTCCGCATTGTCTCCGAAG	1473–1498	Antisense
**NP P**	FAM-AAGGCAACGAACCC-MGB	1421–1435	Sense

Note: The NP and HA primers were 131bp and 178bp, respectively. FP, RP and P respectively represented forward primer, reverse primer and probe for short.

#### Optimization of RRT-PCR

As we used commercial kit (HiScript II U^+^ One Step qRT-PCR Probe Kit, Vazyme, Nanjing, China) in RRT-PCR, the PCR reaction system and programs were provided. So optimization consists of primer design, probe design and concentration adjustment. First, primers and probes were specifically chosen through gene alignments and primer specificity analysis. Then we determined the optimal concentrations of the two probes. Various concentrations of NP and H5-HA probes, i.e., 200nM, 250nM, 300nM and 350nM, were used in every single detection (**Figure A and B in S1 Fig)**. Finally we confirmed the concentrations of HA and NP primers in multiple detection. In this part, amplification curves and dissociation curves (**Figure C and D in S1 Fig**) were performed at different concentrations of HA and NP primer pairs (350-450nM) with SYBR Premix Ex Taq II kit (Takara, Shiga, Japan). The optimal primer and probe concentrations of H5-HA primer pairs, NP primer pairs, H5-HA probe, NP probe in 20 μL RRT-PCR system were 400nM, 400nM, 350nM, 350nM, respectively. A single peak around 82.5°C was obtained in melt curves (**Figure D in S1 Fig**), suggesting no primer-dimer was formed.

#### One-step Real-time PCR

After optimization, primers and probes were ready for PCR amplification. One-step Real-time PCR was performed in a 20μL reaction mixture after optimization. It consisted of 10μL 2x One Step PCR Mix (Vazyme), 1μL Enzyme Mix (Vazyme) containing reverse transcription enzyme and DNA polymerase, 0.35μL H5-HA probe (20μM), 0.35μL NP probe (20μM), 0.4μL H5-HA reverse primer (20μM), 0.4μL H5-HA forward primer (20μM), 0.4μL NP reverse primer (20μM), 0.4μL NP forward primer (20μM), 6μL RNA sample, 0.4μL 50x ROX and 0.3μL RNase-free water. Reactions were carried through in ABI 7500 Real-time PCR instrument (Applied Biosystems) with the following programs: 15min at 55°C, 5min at 95°C, 40 cycles of 5s at 95°C and 34s at 60°C. Meanwhile, NTC (no template control) and positive control (RNA from H5 subtype AIV) were both used. The data was then analyzed with 7500 Software Version 2.0.6 (Applied Biosystems).

#### Specificity, sensitivity and reproducibility of RRT-PCR

When referring to specificity, we firstly used Blastn search engine to test two sets of primers with probes in **Table 2**. Organism option in Blastn was chosen to include every subtype of AIV or exclude AIV before results analysis. Then the specificity was further evaluated using the nucleic acids of H1-H11 subtype AIVs (except H2, **Table 1**) and other avian pathogens including NDV, IBDV, IBV and MDV. And the sensitivity was determined using serially diluted mixture of HA and NP plasmids (each plasmid was 5×10^5^, 5×10^4^, 5×10^3^, 5×10^2^, 5×10^1^, 5×10^0^ copies of DNA/ 5 μL) as quantitative standards for the calculation of copy number. Furthermore, for RNA samples, serial logarithmic dilutions of RNA according to EID_50_ were used for amplification by pan-AIV or H5 RRT-PCR. To test the reproducibility in our study, inter-assays and intra-assays were analyzed by diluting plasmids mixture to 4 different concentrations. In intra-assays, triplicates were made in multiplex RRT-PCR on one plate while in inter-assays we repeated multiplex RRT-PCR on day 1, day 3, and day 5.

### Experimental and clinical samples

#### Experimentally inoculated oral-pharyngeal and cloacal swabs

Three of ten SPF chickens were infected intranasally (i.n.) with 10^3.0^ EID_50_ of H5 AIV strain 0420 (**Table 1**). After inoculation, all the chickens were kept in one isolator for mimicking natural infections (n = 10). From 24 hours post infection (p.i.) to 48 h p.i., the oral-pharyngeal swab and cloacal swab of each chicken were collected in 1 mL antibiotics-containing PBS. Samples were stored at -70°C until total nucleic acids were extracted with High Pure Viral Nucleic Acid Kit (Roche Molecular Biochemicals).

#### Clinical cloacal swab samples collection

Oral-pharyngeal and cloacal swab samples were collected from live poultry market of East China. Swab samples were immediately placed into 1mL antibiotic-containing PBS as described above and then stored at -70°C until total nucleic acids were extracted with viral RNA/DNA extraction kit above.

#### Virus isolation and identification

After collecting swab samples from experimentally inoculated chickens and live poultry markets, 200μL volume was used for viral nucleic acid extraction followed by RRT-PCR. Meanwhile, another 200μL was used for traditional virus isolation using 10-day-old embryonated SPF chicken eggs. After 3 days of incubation at 35°C, the presence of virus was determined by the hemagglutination assay using 1% chicken erythrocytes. In addition, the specific information of subtypes collected from market was further confirmed by hemagglutination inhibition test and PCR with AIV universal primers [36].

## Results

### Specificity of H5 and pan-AIV RRT-PCR

The primers and probes were obtained through gene alignment and primer design. The results of Blastn were shown in **S1 Table** and **S2 Table**. As shown in **S1 Table,** there were 20333 results which matched primers and single probe targeting NP gene, suggesting the potential of this group of primers and single probe to detect pan-AIVs from H1 to H16. Similarly, 7598 results that matched primers and single probe targeting H5-HA gene were found in **S2 Table**, indicating the possibility of the primers and single probe to detect H5N1 to H5N9. The results showed that all 9 tested AIV subtypes as well as different clades of H5 could be specifically detected (**Table 1, S2–S4 Figs**). In contrast, NDV, IBDV, IBV and MDV as the negative controls could not be amplified with primers and probes in **Table 2**.

### Standard curves for multiplex one-step RRT-PCR

**Fig 1:**
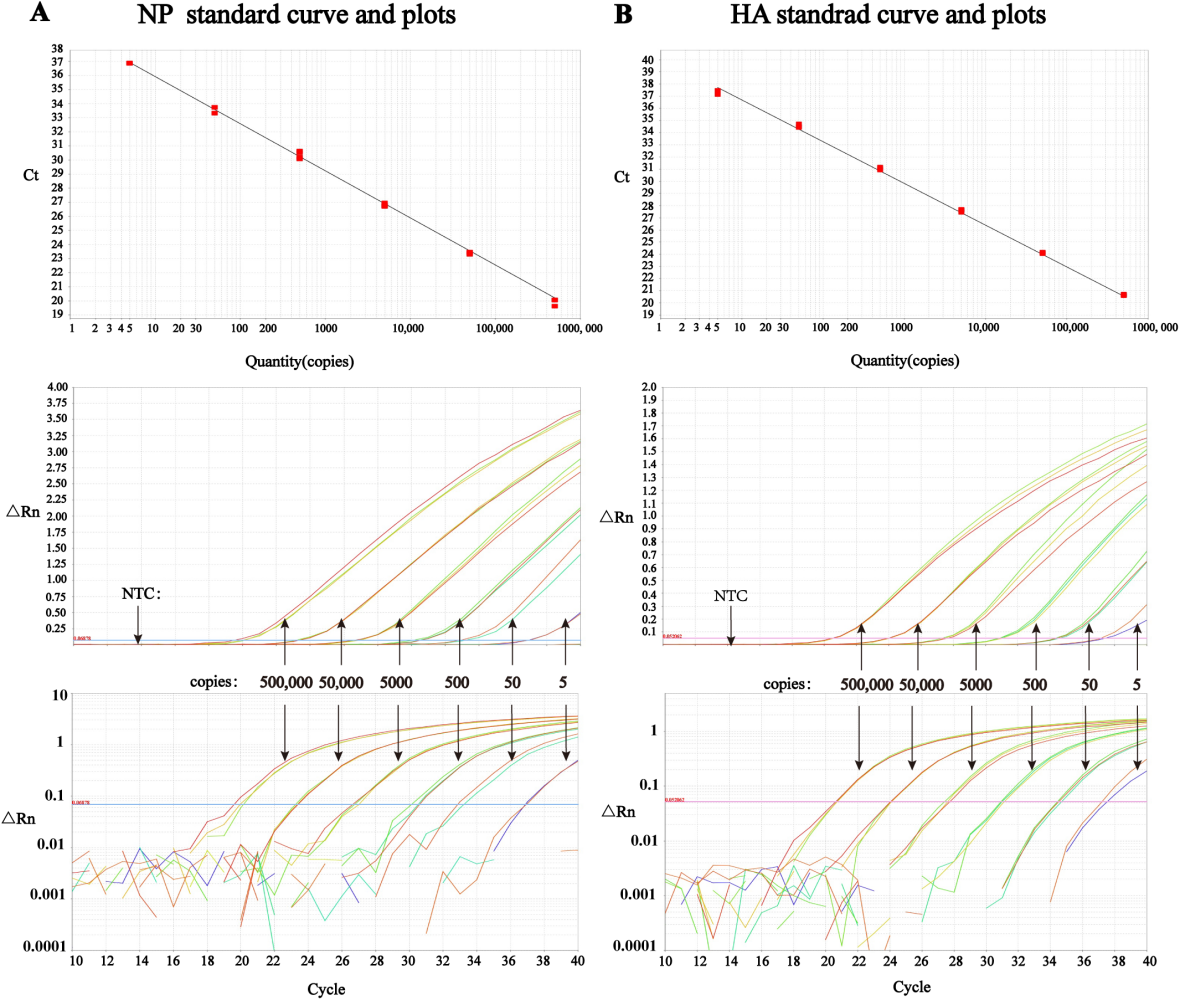
Amplification plots and standard curves drew by ABI 7500 software in multiplex detection with standard plasmids. The threshold lines in amplification plots and linear curves in standard curves were draw automatically by ABI 7500 software. (A) NP standard curve between 5x10^0^ and 5x10^5^ copies (y = -3.346x+39.272, R^2^ = 0.997, top), linear amplification plot (middle), and log amplification plot (bottom) in multiplex detection. (B) HA standard curve between 5x10^0^ and 5x10^5^ copies (y = -3.43x+40.183, R^2^ = 0.998, top), linear amplification plot (middle), and log amplification plot (bottom) in multiplex detection.

In order to determine the detection limit of our method, two standard plasmids which carried H5-HA gene and NP gene were constructed respectively. Standard curves were of linear correlation between 5x10^5^ and 5x10^0^ copies of the target DNAs in multiplex detection (**Fig 1**). In addition, the linear relation expresses of standard curves of NP and HA were y = -3.346x+39.272 (Efficiency 99.01%, R^2^ 0.997) and y = -3.43x+40.183 (Efficiency 95.67%, R^2^ 0.998), respectively. Moreover, the multiplex detection limit of H5-AIV and pan-AIV RRT-PCR was as low as 5 copies per reaction (or 5 copies/μL).

### Detection limits of RNA samples

The detection limit of different subtypes of AIVs and different clades of H5 were tested. As for RRT-PCR for pan-AIV detection, the detection limit of different HA subtypes was between 0.021 to 1.0 EID_50_ (**Table 1, S2 Fig and S3 Fig**), suggesting a better sensitivity than virus isolation. While in H5 detection, the detection limit of different clades was from 0.021 to 0.1EID_50_ (**S4 Fig and Table 1**).

### Reproducibility assays

The results of intra-assays (**Table 3**) and inter-assays (**Table 4**) of AIV-NP and H5-HA detection revealed that the coefficients of variation (CV %) were all < 1%, suggesting our multiplex RRT-PCR method is of high reproducibility considering a threshold of 3% for CV % [37, 38].

**Table 3:** Intra-assay in multiplex detection of H5 AIV and pan-AIV.

Copies of plasmids	Gene	Ct Value in Intra-Assay			Mean ± SD	CV%
		1	2	3		
**5x10**^**4**^	HA	22.899	23.113	23.013	23.014±0.108	0.469%
	NP	22.356	22.239	22.309	22.301±0.059	0.264%
**5x10**^**3**^	HA	26.558	26.658	26.686	26.634±0.067	0.253%
	NP	25.770	25.748	25.835	25.784±0.045	0.175%
**5x10**^**2**^	HA	29.982	29.982	30.094	30.019±0.065	0.215%
	NP	29.231	29.280	29.237	29.249±0.027	0.091%
**5x10**^**1**^	HA	32.759	32.927	32.946	32.878±0.103	0.313%
	NP	32.280	32.561	32.561	32.373±0.163	0.503%

Note: Each concentration had 3 replicates. CV% (the threshold is 3%) and Ct values of each concentration were shown.

**Table 4:** Inter-assay in multiplex detection of H5 AIV and pan-AIV.

Copies of plasmids	Gene	Ct Value in Inter-Assay			Mean ± SD	CV%
		Day1	Day3	Day5		
**5x10**^**5**^	HA	18.319	18.239	18.332	18.297±0.050	0.274%
	NP	20.153	20.332	20.139	20.208±0.107	0.532%
**5x10**^**4**^	HA	22.085	21.914	21.968	21.989±0.087	0.397%
	NP	22.999	23.088	22.988	23.025±0.055	0.239%
**5x10**^**3**^	HA	25.039	24.745	24.866	24.883±0.147	0.592%
	NP	26.306	26.527	26.533	26.455±0.130	0.490%
**5x10**^**2**^	HA	28.841	28.368	28.701	28.637±0.243	0.848%
	NP	30.157	30.569	30.056	30.405±0.302	0.897%

Note: We repeated multiplex RT-PCR on day 1, day 3, and day 5. CV% (the threshold is 3%) and Ct values of each concentration were shown.

### Comparison of RRT-PCR and virus isolation for AIV detection in experimental samples

**Fig 2:**
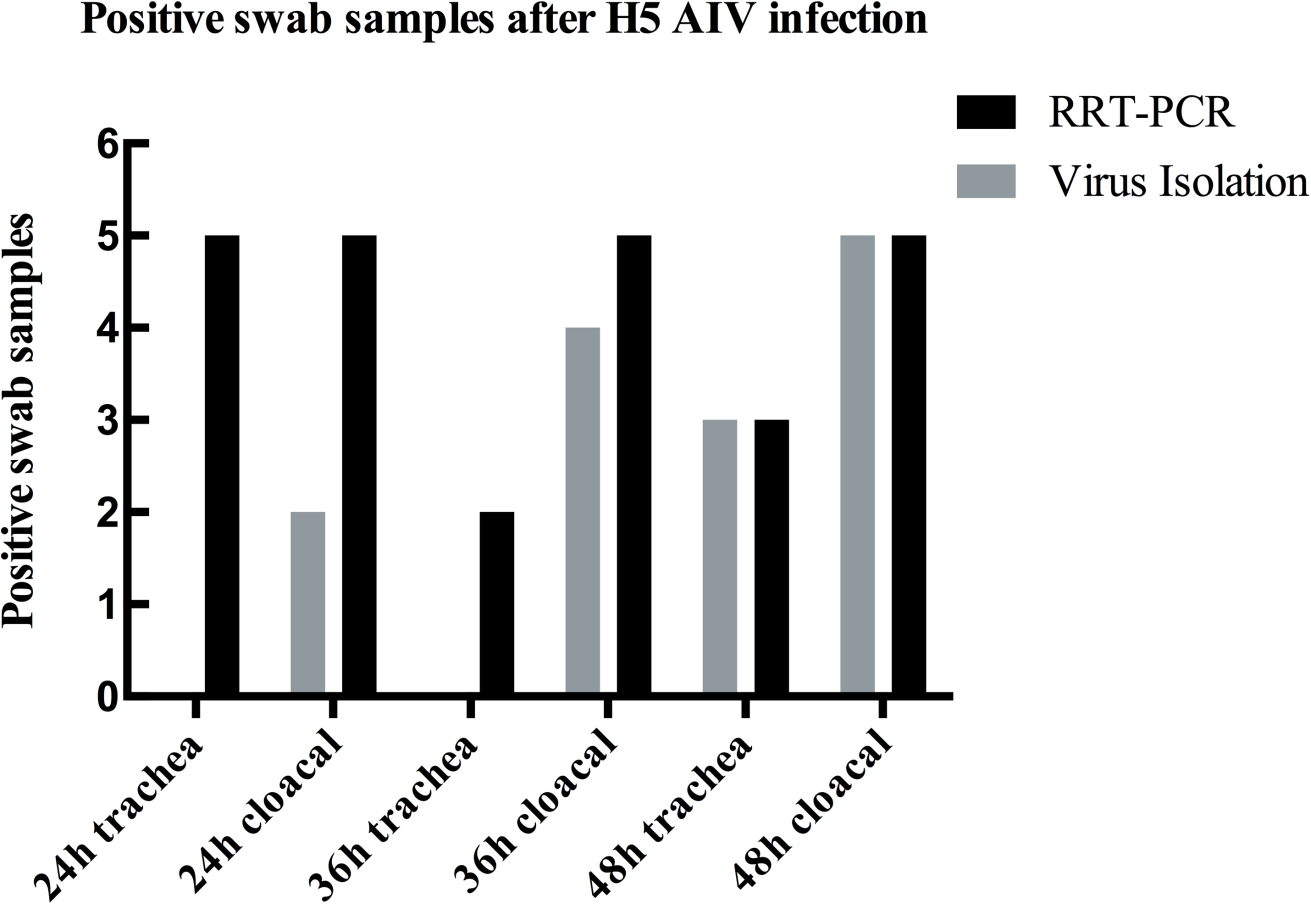
Detection of H5 AIV in swabs from infected SPF chickens with virus isolation and RRT-PCR. Oral-pharyngeal swabs and cloacal swabs of 10 SPF chickens were collected at 24 h, 36 h and 48 h. The infection dose of SPF chicken was 0.2mL 10^3^ EID_50_ of H5 AIV. At 24 h and 36 h, no H5 AIV was detected in oral-pharyngeal samples in traditional virus isolation method while the number of positive trachea swabs through RRT-PCR detection at 24 h and 36 h were 5 and 2 respectively. The number of positive samples in RRT-PCR matched that in virus isolation at 48 h.

H5 AIV usually kills the infected chickens in 3 days, so we collected swabs at 24 h, 36 h and 48 h post infection. Three 6-weeks-old SPF chickens (n = 10) were inoculated i.n. with 0.2 ml 10^3^ EID_50_ H5 subtype AIV and another 7 chickens of the group were kept contact with these infected chickens. Duplicates were made for each sample in RRT-PCR and the criterion of positive sample was Ct of duplicates less than 40 in both FAM and VIC fluorescence channels. As shown in **Fig 2**, the number of positive samples of each column in RRT-PCR was higher than that of virus isolation method, except the time point of 48 h at which positive samples of two methods were equal in number.

### Comparison of RRT-PCR and virus isolation for AIV detection in clinical samples

**Fig 3:**
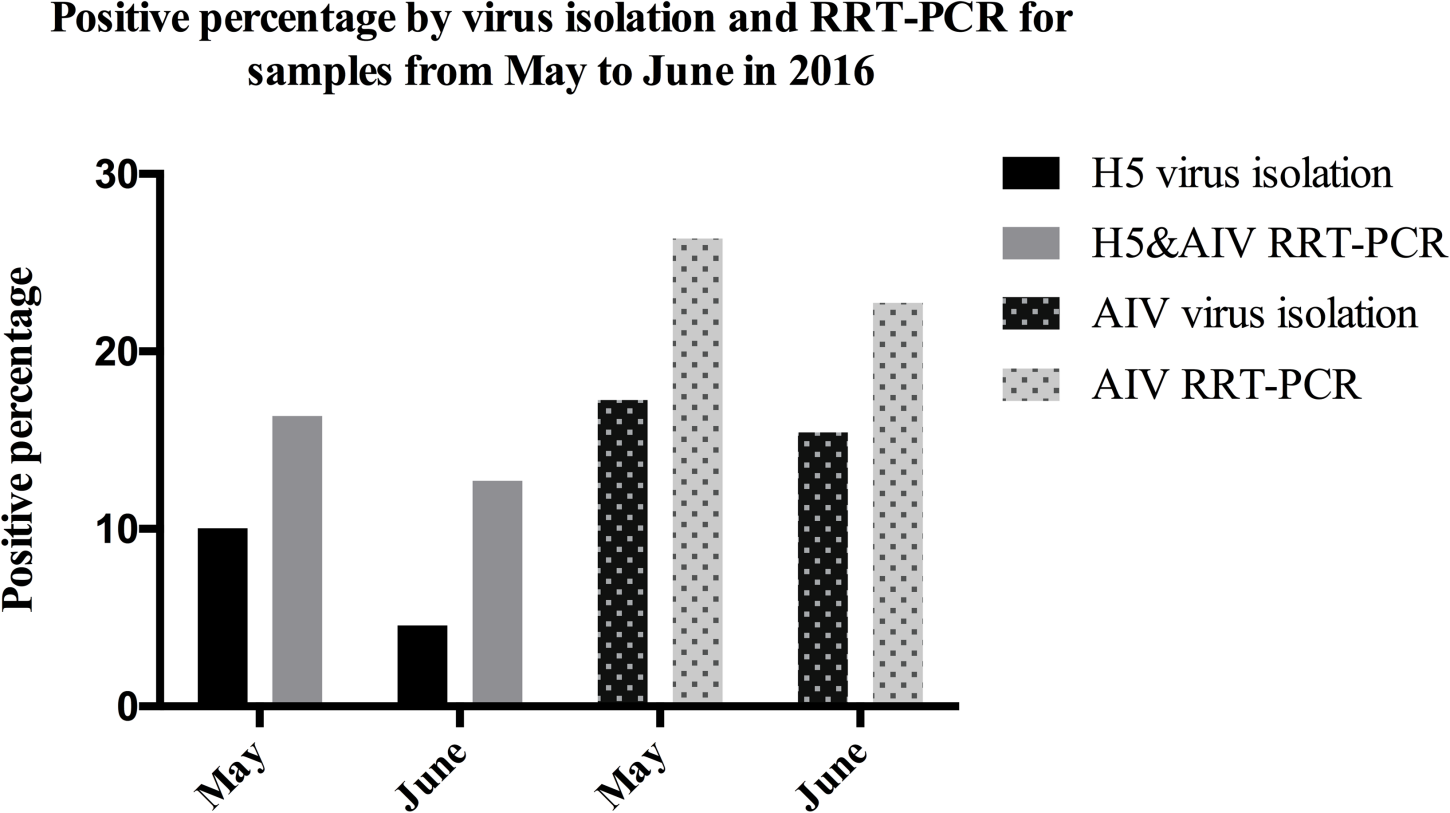
Positive percentage by virus isolation and RRT-PCR for live-poultry market samples from May 2016 to June 2016. 110 oral-pharyngeal or cloacal samples were collected from live poultry market in May and June respectively in 2016. Columns from left to right are 10%, 16.4%, 4.5%, 12.7%, 17.3%, 26.4%, 15.5%, 22.7%, which showed RRT-PCR method was of higher positive percentage than virus isolation.

Oral-pharyngeal or cloacal swab samples were collected in May and June from live poultry market of East China. All these samples were tested simultaneously by RRT-PCR and virus isolation methods. Meanwhile, after HA test, positive samples in virus isolation method were further classified into H5 and other subtypes through HA-HAI test and regular RT-PCR with AIV universal primers. The result revealed that our RRT-PCR method was more sensitive than virus isolation. Specifically, 36 (16.36% of 220) AIV strains containing 16 (7.27% of 220) H5 strains in these two months were collected via virus isolation in eggs (**Fig 3**), while 54 (24.55% of 220) samples were AIV positive and 32 (14.54% of 220) samples were H5 positive by RRT-PCR method.

## Discussion

The frequent antigen shift and antigen drift of AIV increase the difficulty of AIV detection. Among all the AIV subtypes, highly pathogenic H5 avian influenza virus often leads to high morbidity and mortality in poultry. Therefore, early detection of AIV and H5 subtype is very necessary in the surveillance and control of AIV outbreaks. RRT-PCR is an effective detection method and multiplex detection is an inevitable tendency in early detection because of its high efficiency and reproducibility. When referring to RRT-PCR method based on MGB, it has multiple advantages, including keeping high Tm, shortening the length of probe [30, 39], lowering the fluorescence background [40] and helping the combination of probe and template [41]. Although some Taqman-MGB detection methods for H5 AIV have been reported before 2010 [39, 42], it is of high necessity that the sensitivity and specificity could be further developed owing to the high variation of influenza virus.

Among AIV detection methods, Teng [43] reported the detection of H3 AIV through Taqman-MGB method, with detection limit of 10 copies per reaction, which was 1000 times more sensitive than conventional RT-PCR. Di [44] also found that the sensitivity of M gene Taqman-MGB probe for pan-AIV detection was 10–100 times higher than conventional RT-PCR which was between 5–50 RNA copies per reaction. Payungporn [39] designed MGB probes for M, NA and HA gene of H5N1 in 2006 and the LOD (limit of detection) of the multiplex RT-PCR was 100 copies/uL. In 2007, Lu [42] designed Taqman-MGB probe targeting H5-HA for the detection of H5 subtype and the detection threshold was 100 copies per reaction. Although these Taqman-MGB detection methods for H5 AIV have been reported before 2010, there is possibility that the sensitivity could be further developed. Moreover, none of them have reported suitable Taqman probes for the detection of different clades of H5 AIV.

In our study, Taqman-MGB probes and primers were designed based on the conserved regions of H5-HA gene and AIV-NP gene. High specificity and repeatability of the AIV-NP and H5-HA RRT-PCR was successfully achieved. As low as 0.021~1 EID_50_ AIV per reaction or 5 copies/μL (or 5 copies per reaction) nucleic acids of NP and H5-HA gene could be detected, indicating the potential value of our method in application of early detection and rapid diagnosis of AIV. In the detection of experimental and clinical samples, our multiplex RRT-PCR method showed higher sensitivity along with high efficiency. To the best of our knowledge, this is the first Taqman-MGB method for the detection of multiple H5 clades and the first report of NP Taqman-MGB probe for pan-AIV detection although NP is also a very conserved gene except for M gene in AIV [45].

In conclusion, the multiplex RRT-PCR method established in our study showed high sensitivity, reproducibility and specificity. As our RRT-PCR provides a fast and convenient method for screening samples in large volumes during clinical diagnose, it can be applied before traditional virus isolation method which was used to verify and isolate AIV strains. It is expected that this multiplex RRT-PCR for detection of pan-AIV and its H5 subtype will be applied in surveillance of clinical samples in live poultry markets.

## Supporting information

S1 Fig Optimization of oligonucleotide concentrations. **(A)** Curves representing fluorescence and Ct values for H5-HA primers using 200, 250, 300 or 350nM in reaction. **(B)** Curves representing fluorescence and Ct values for NP primers using 200, 250, 300 or 350nM in reaction. **(C)** Curves comparing different concentration sets of H5-HA and NP primers in SYBR Green PCR, i.e., NP350 and HA350nM, NP400 and HA350nM, NP400 and HA400nM, NP400 and HA450nM. **(D)** Post-PCR melting curves displaying fluorescence versus temperature in SYBR Green PCR.
(TIF)

S2 Fig Amplification plot of diluted RNA of H1, H3, H4, H6, H7, H8, H9, H10, H11 AIVs in AIV-NP RRT-PCR with curves of other avian pathogens as negative controls. After extraction, RNAs of different subtypes of AIVs were diluted logarithmically according to EID_50_. E.g., The Ct values of H1N1 varies from 21 to 35 when RNA was diluted to 4200 ~ 0.042 EID_50_.
(TIF)

S3 Fig Amplification plot of diluted RNA of H5 clades in AIV-NP RRT-PCR with curves of other avian pathogens as negative controls. (TIF)

S4 Fig The detection limit of different clades of H5 subtype AIV tested in H5-HA RRT-PCR. Amplification plot of diluted RNA of H5 clades in H5-HA RRT-PCR, with curves of other avian pathogens as negative controls. RNAs were diluted logarithmically according to EID_50_. E.g., The Ct values of SQ1404 varies from 21 to 38 when RNA was diluted to 4200 ~ 0.042 EID_50_.
(TIF)

S1 Table Number of items of Blastn after inputting NP primers and probes for detecting pan-AIV. (DOC)

S2 Table Number of items of Blastn after inputting HA primers and probes for detecting all H5 AIV. (DOC)
