# Supplementary figures and images for: Multiplex one-step Real-time PCR by Taqman-MGB method for rapid detection of pan and H5 subtype avian influenza viruses
Source: PLoS One. 2017 Jun 2;12(6):e0178634. doi: 10.1371/journal.pone.0178634 (PMC5456101; doi:10.1371/journal.pone.0178634)

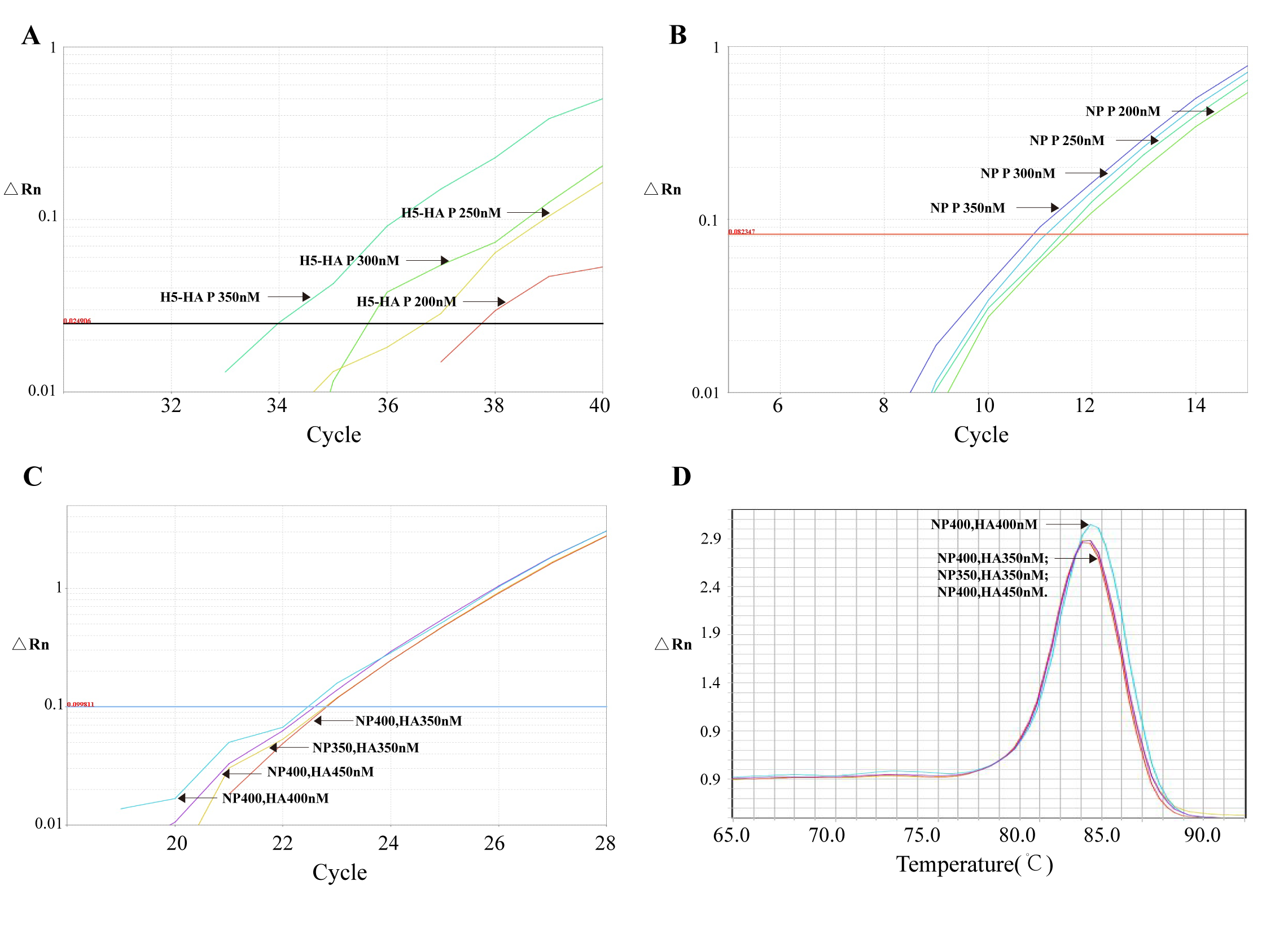

Supplement: S1 Fig — (A) Curves representing fluorescence and Ct values for H5-HA primers using 200, 250, 300 or 350nM in reaction. (B) Curves representing fluorescence and Ct values for NP primers using 200, 250, 300 or 350nM in reaction. (C) Curves comparing different concentration sets of H5-HA and NP primers in SYBR Green PCR, i.e., NP350 and HA350nM, NP400 and HA350nM, NP400 and HA400nM, NP400 and HA450nM. (D) Post-PCR melting curves displaying fluorescence versus temperature in SYBR Green PCR. (TIF) [file pone.0178634.s001.tif]

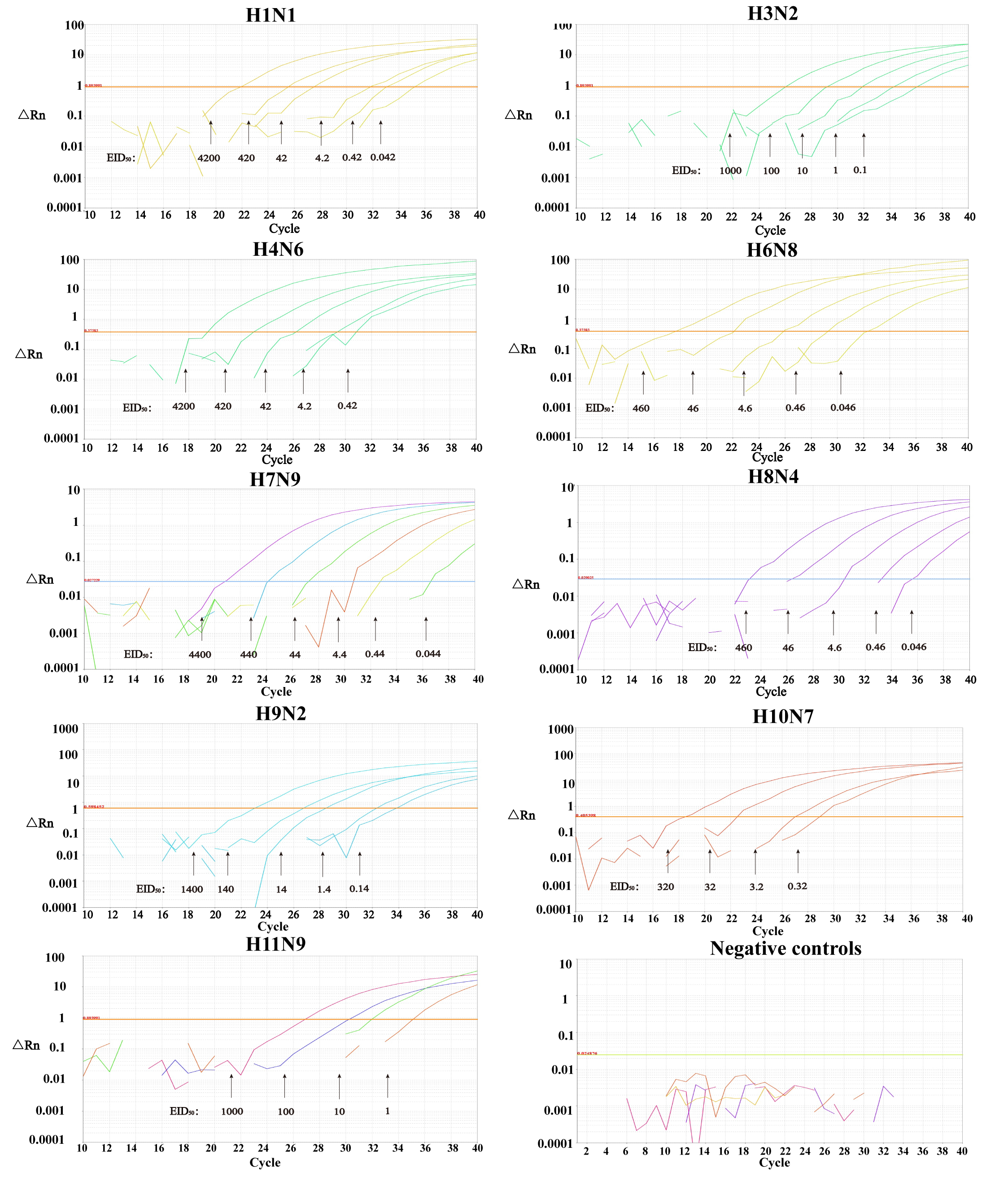

Supplement: S2 Fig — After extraction, RNAs of different subtypes of AIVs were diluted logarithmically according to EID50. E.g., The Ct values of H1N1 varies from 21 to 35 when RNA was diluted to 4200 ~ 0.042 EID50. (TIF) [file pone.0178634.s002.tif]

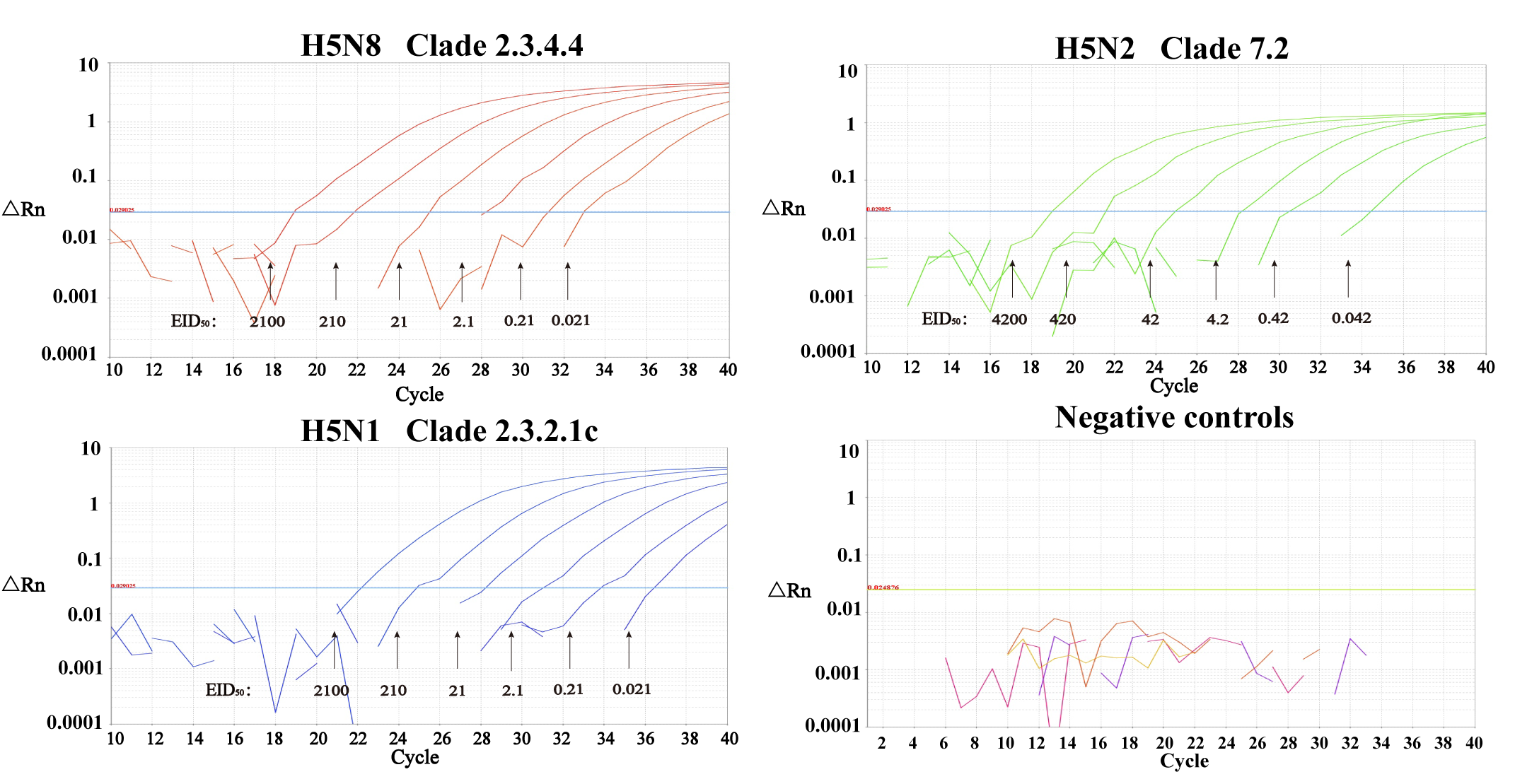

Supplement: S3 Fig — (TIF) [file pone.0178634.s003.tif]

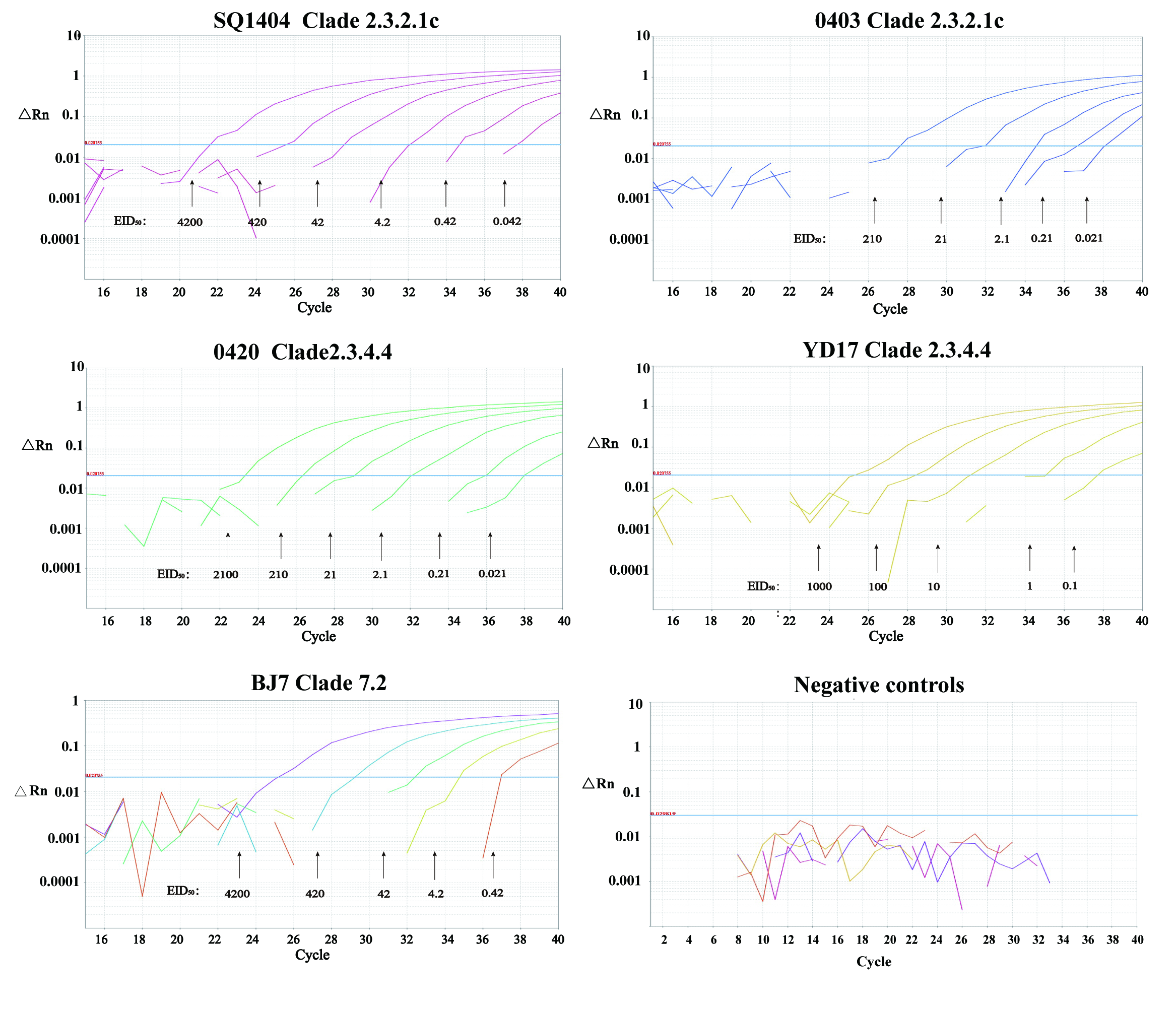

Supplement: S4 Fig — Amplification plot of diluted RNA of H5 clades in H5-HA RRT-PCR, with curves of other avian pathogens as negative controls. RNAs were diluted logarithmically according to EID50. E.g., The Ct values of SQ1404 varies from 21 to 38 when RNA was diluted to 4200 ~ 0.042 EID50. (TIF) [file pone.0178634.s004.tif]
