# Supplementary material for: Multiplex one-step Real-time PCR by Taqman-MGB method for rapid detection of pan and H5 subtype avian influenza viruses
Source: PLoS One. 2017 Jun 2;12(6):e0178634. doi: 10.1371/journal.pone.0178634 (PMC5456101; doi:10.1371/journal.pone.0178634)
Supplement: S1 Table — (DOC) [file pone.0178634.s005.doc]

**S1 Table. Number of items of Blastn after inputting NP primers and probes for detecting pan-AIV.**

|  | **H1** | **H2** | **H3** | **H4** | **H5** | **H6** | **H7** | **H8** | **H9** | **H10** | **H11** | **H12** | **H13** | **H14** | **H15** | **H16** | **Total** |
| --- | --- | --- | --- | --- | --- | --- | --- | --- | --- | --- | --- | --- | --- | --- | --- | --- | --- |
| **N1** | 4421 | 41 | 53 | 10 | 1924 | 255 | 137 | 1 | 46 | 24 | 28 | 5 | 0 | 0 | 0 | 0 | 6945 |
| **N2** | 803 | 175 | 4216 | 143 | 569 | 425 | 159 | 2 | 1191 | 21 | 83 | 3 | 31 | 2 | 2 | 0 | 7825 |
| **N3** | 30 | 143 | 20 | 18 | 88 | 10 | 341 | 1 | 4 | 50 | 23 | 5 | 11 | 1 | 0 | 84 | 829 |
| **N4** | 8 | 5 | 1 | 21 | 3 | 10 | 21 | 95 | 0 | 33 | 1 | 8 | 0 | 0 | 4 | 0 | 210 |
| **N5** | 7 | 17 | 12 | 14 | 12 | 55 | 3 | 0 | 13 | 18 | 4 | 102 | 0 | 4 | 0 | 0 | 261 |
| **N6** | 19 | 4 | 214 | 587 | 87 | 172 | 30 | 0 | 4 | 26 | 14 | 6 | 57 | 6 | 5 | 0 | 1231 |
| **N7** | 3 | 24 | 9 | 5 | 171 | 3 | 189 | 0 | 5 | 323 | 9 | 5 | 0 | 0 | 2 | 0 | 748 |
| **N8** | 18 | 10 | 879 | 136 | 82 | 147 | 12 | 1 | 5 | 53 | 17 | 4 | 11 | 2 | 1 | 0 | 1378 |
| **N9** | 27 | 55 | 7 | 11 | 21 | 10 | 467 | 0 | 10 | 21 | 257 | 4 | 13 | 0 | 3 | 0 | 906 |
| **Total** | 5336 | 474 | 5411 | 945 | 2957 | 1087 | 1359 | 100 | 1278 | 569 | 436 | 142 | 123 | 15 | 17 | 84 | 20333 |

Note: Ident > 80%, quenry cover > 80%.
