# Supplementary material for: Multiplex one-step Real-time PCR by Taqman-MGB method for rapid detection of pan and H5 subtype avian influenza viruses
Source: PLoS One. 2017 Jun 2;12(6):e0178634. doi: 10.1371/journal.pone.0178634 (PMC5456101; doi:10.1371/journal.pone.0178634)
Supplement: S2 Table — (DOC) [file pone.0178634.s006.doc]

**S2 Table. Number of items of Blastn after inputting HA primers and probes for detecting all H5 AIV.**

| **NA Subtype** | **BLAST Results** |
| --- | --- |
| H5N1 | 6013 |
| H5N2 | 1184 |
| H5N3 | 100 |
| H5N4 | 10 |
| H5N5 | 33 |
| H5N6 | 105 |
| H5N7 | 13 |
| H5N8 | 111 |
| H5N9 | 29 |
| Total | 7598 |

Note: Ident>80%, quenry cover>80%.
